# Supplementary material for: Necroptosis Blockade Potentiates the Neuroprotective Effect of Hypothermia in Neonatal Hypoxic-Ischemic Encephalopathy
Source: Biomedicines. 2022 Nov 13;10(11):2913. doi: 10.3390/biomedicines10112913 (PMC9687213; doi:10.3390/biomedicines10112913)
Supplement: Supplementary file 1 [file biomedicines-10-02913-s001.zip › biomedicines-1998711-supplementary.pdf]

Supplementary table S1. List and features of antibodies

Antibody	Company-reference number	Dilution
Rabbit anti-rat TNF-α polyclonal antibody	Sigma-Aldrich (AB1837P)	1/100
Rabbit anti-RIP3 (phospo S232) monoclonal antibody	Abcam (ab195117)	1/50
Rabbit anti-MLKL (phosphor S345) monoclonal antibody	Abcam (ab 196436)	1/50
Goat anti rabbit IgG-HRP	Santa cruz (sc-2004)	1/100
